# Self‐Reported Adverse Events in Psychiatric Outpatients Taking Hypnotics: Higher Real‐World Frequencies Than Those Listed in Package Inserts

**DOI:** 10.1002/npr2.70162

**Published:** 2026-08-28

**Authors:** Fumi Akasaka, Kuni Akasaka, Kazutake Okada, Naohito Yamaguchi, Yasuo Haruki, Sotaro Sadahiro

**Affiliations:** ^1^ Akebono Clinic Ishinomaki Japan; ^2^ Department of Surgery Tokai University Hospital Isehara Japan; ^3^ Research Division Saiseikai Research Institute of Health Care and Welfare Minatoku Japan; ^4^ Department of Basic Medical Science Tokai University Hospital Isehara Japan

**Keywords:** adverse events, benzodiazepines, dual orexin receptor antagonists, hypnotics, non‐benzodiazepines

## Abstract

**Introduction:**

Benzodiazepines (BZDs) are associated with dependence, tolerance, withdrawal, and various adverse effects, and switching to other drug classes is often recommended. Although non‐BZD hypnotics are reported in package inserts to have similar but less frequent adverse events, real‐world patients often have complex psychiatric and medical backgrounds, and the actual frequency of subjective adverse events may differ substantially from that reported in package inserts. This study aimed to elucidate subjective symptoms potentially related to psychotropic medications using real‐world patient data.

**Methods:**

This prospective cohort study included patients at the Akebono Clinic who were taking a single hypnotic agent. A total of 314 patients were analyzed. Insomnia severity was assessed using the Athens Insomnia Scale, and 11 self‐reported adverse events were evaluated.

**Results:**

The frequency of insomnia was 72% for non‐BZDs, 52% for BZDs, and 70% for dual orexin receptor antagonists (DORAs) (significantly lower for BZDs; *p* = 0.0036). Among the 11 self‐reported symptoms, two items (thirst and nightmares) were reported as “always” or “often” by more than 10% of patients; three items (constipation, head heaviness/headache, and abnormal behavior during sleep) by 5%–10%; and four items (lightheadedness, vertigo, abdominal pain, and tremor) by 3%–5%. There were no differences in adverse‐event frequencies among non‐BZDs, BZDs, and DORAs. Multivariate analyses showed that adverse events were associated with female sex, age < 60 years, and schizophrenia, depression, and anxiety disorders, but not with hypnotic drug classes.

**Conclusions:**

Subjective adverse events in patients taking hypnotics occurred more frequently than those reported in package inserts, indicating a clear discrepancy between controlled trial data and real‐world clinical experience. Because adverse events were more strongly associated with patient characteristics—such as psychiatric comorbidities, physical conditions, and concomitant medications—than with hypnotic drug class, routine assessment of subjective symptoms through interviews or questionnaires is essential in clinical practice.

## Abstract 2

Subjective adverse events in patients taking hypnotics occurred more frequently than those reported in package inserts, indicating a clear discrepancy between controlled trial data and real‐world clinical experience. Adverse events were more strongly associated with patient characteristics—such as psychiatric comorbidities, physical conditions, and concomitant medications—than with hypnotic drug class.

## Graphical abstract

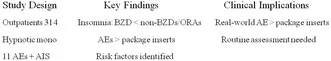

## 1 Introduction

Sleep is an essential activity for the proper functioning of the brain, occupying approximately one‐third of our lifetime. In addition, many reports suggest that sleep plays an important role in the recovery from fatigue, maintenance of homeostasis, and regulation of body temperature [1]. According to Japanese Government Statistics in 2016, 23.2% of the general population reported that they do not get enough rest through sleep, with insomnia and inadequate sleep being major problems among the general population [2]. Currently, non‐benzodiazepines (non‐BZDs), benzodiazepines (BZDs), the relatively new dual orexin receptor antagonists (DORAs) [3], and melatonin receptor agonists are mainly used as sleep medications for insomnia.

BZDs are associated with dependence, tolerance, withdrawal, and adverse effects such as lightheadedness and cognitive dysfunction; therefore, switching to a different drug class is recommended [4, 5]. On the other hand, it has been reported that non‐BZD hypnotics have the same risks of falls, fractures, and cognitive dysfunction as BZD hypnotics [4, 6, 7, 8]. In addition, the U.S. Food and Drug Administration (FDA) recently reported that non‐BZD hypnotics significantly increase bone joint, oral cavity, respiratory system, and urinary tract infections [9]. A recent systematic review found no high‐ or moderate‐certainty evidence for any method of discontinuing BZDs or closely related sedative‐hypnotics [10].

In a survey of hypnotic‐related adverse events reported by the University of Chicago and Cleveland Clinic, the frequency was as low as 0.01% or less [11]. The incidence of adverse events listed in the package inserts or prescribing information for each drug has also been reported to be low. However, real‐world psychiatric outpatients often have multiple comorbidities, polypharmacy, and chronic use of hypnotics, making their clinical background far more complex than the populations reflected in package‐insert data. This discrepancy suggests that package‐insert frequencies may substantially underestimate the actual burden of adverse events experienced by patients in routine clinical settings. Contrarily, in clinical practice, the frequency of subjective adverse events reported by psychiatric outpatients taking hypnotics is high [12].

Regarding the efficacy and safety of hypnotics, non‐BZDs have been found to be less effective in older adults [13], whereas daridorexant, a DORA, shows no difference in efficacy and safety between older and younger adults [14]. It has been reported that the peak concentration of zolpidem is 45% higher in women than in men [15]. Therefore, there may be age‐ and sex‐related differences in associated efficacy and adverse events.

In the present study, we investigated the subjective symptoms potentially related to psychotropic medications, such as lightheadedness, vertigo, headache, nausea, constipation, dysgeusia, thirst, and nightmares, in addition to sleep satisfaction, to obtain useful information when changing from BZDs to other drug classes in clinical practice.

## 2 Materials and Methods

### 2.1 Study Design and Participants

This study was a prospective cohort study. Patients who visited the Akebono Clinic between November 2023 and October 2024 and were taking hypnotics were included. Patients with severe mental retardation, severe dementia, or significant hallucinatory and delusional states were excluded because they could not complete the questionnaire sufficiently or respond appropriately during interviews. Overall, 506 patients were included in the study. To minimize confounding by hypnotic polypharmacy, only patients receiving a single hypnotic agent were included in the analysis, resulting in 314 eligible participants. Because this study was conducted in a real‐world psychiatric outpatient setting, many patients had multiple comorbidities and were receiving several psychotropic medications.

### 2.2 Data Collection

Sex, age, psychiatric diagnoses according to the DSM‐5 [16, 17], and the type and number of psychotropic drugs that the patient was taking were obtained from their medical charts. If there was a comorbid psychiatric disorder, the primary psychiatric diagnosis was noted. Psychotropic medications were classified into the following categories: hypnotics, antipsychotics, anxiolytics, antidepressants, and mood stabilizers. Hypnotics were classified into four drug classes: non‐BZDs, BZDs, DORAs, and melatonin receptor agonists.

### 2.3 Self‐Assessment of Insomnia

For the self‐assessment of insomnia, we used the Japanese version of the Athens Insomnia Scale (AIS) [18, 19], which was self‐administered in the waiting room before consultation. The questionnaire was administered only when the same hypnotic had been prescribed for at least 1 month to ensure stable drug effects. Each item is rated on a 0–3 scale, with higher scores indicating more severe symptoms. According to the classification by Okajima et al., patients with a total score of 6 or more were defined as having insomnia [18].

### 2.4 Survey of Subjective Symptoms and Adverse Events

We investigated 11 subjective adverse events in this study. These events were selected because they are listed as common or clinically important adverse reactions in the Japanese package inserts of the hypnotics. The following 11 adverse events were assessed using a questionnaire: lightheadedness, vertigo, head heaviness/headache, nausea, constipation, abdominal pain, dysgeusia, thirst, nightmares, tremors, and abnormal sleeping behavior. Abnormal sleeping behavior, similar to complex behavior, was evaluated based on reports from family members or cohabitants [20]. The frequency of occurrence was rated on a 3‐point scale: “always or often,” “occasional,” and “none.”

Detailed item‐level frequencies and raw response distributions for all 11 subjective adverse events are provided in the Supporting Information.

### 2.5 Statistical Analyses

Correlations between insomnia and hypnotic drug classes, subjective adverse events and drug classes, and subjective adverse events and concomitant medications were assessed using Fisher's exact test. Subjective adverse events were dichotomized as “always or often” versus “occasional or none.”

To examine the effects of sex, age, psychiatric diagnoses, and hypnotic drug class on adverse events (0 = no/occasional, 1 = yes/always or often), binomial logistic regression was conducted using maximum likelihood estimation. Age was categorized into three groups (< 40, 40–59, and ≥ 60 years), with ≥ 60 years as the reference. Male sex, sleep–wake disorders, and non‐BZDs were used as reference categories. Because psychiatric diagnoses and concomitant psychotropic medications are strongly correlated, concomitant medications were not included in the model to avoid multicollinearity. Six psychiatric diagnoses with 20 or more cases were included (Table 1), resulting in 287 participants in the regression analysis. Data were analyzed using R software (version 4.3.1).

**TABLE 1:** Clinical characteristics of patients (*n* = 314).

Sex	
Male	118
Female	196
Age (years old, median [25%, 75%])	50 [37–65]
< 40 years old	96
40–59 years old	115
≧ 60 years old	103
DSM‐5‐TM classification	
Depressive disorders (DDs)	134
Anxiety disorders (ADs)	52
Sleep–wake disorders (SWDs)	33
Neurodevelopmental disorders (NDDs)	30
Trauma‐ and stress‐related disorders (PTSDs)	21
Schizophrenia (SP)	20
Other disorders	24
Hypnotics	
Non‐benzodiazepines (non‐BZDs)	88
Benzodiazepines (BZDs)	106
Orexin receptor antagonists (ORAs)	117
Melatonin receptor agonist	3
Concomitant medication	
Antidepressants (ADs)	195
Anxiolytics (ALs)	130
Antipsychotics (APs)	47
Mood stabilizers (MSs)	26
No concomitant medicine user	58

## 3 Results

### 3.1 Patient Characteristics (Table 1)

Among the 314 patients included in the analysis, 118 were men and 196 were women. The median age was 50 years (range: 17–97 years). Ninety‐six patients were under 40 years old, 115 were between 40 and 59 years old, and 103 were 60 years or older. Psychiatric disorders with 20 or more patients included depressive disorders (*n* = 134), anxiety disorders (*n* = 52), sleep–wake disorders (*n* = 33), neurodevelopmental disorders (*n* = 30), trauma‐ and stress‐related disorders (*n* = 21), and schizophrenia (*n* = 20). Non‐BZDs were used in 88 patients (28%), BZDs in 106 (34%), DORAs in 117 (37%), and melatonin receptor agonists in three (1%). Because only three patients were taking a melatonin receptor agonist, comparisons were made among non‐BZDs, BZDs, and DORAs. Concomitant medications included antidepressants in 195 patients, anxiolytics in 130, antipsychotics in 47, and mood stabilizers in 26.

### 3.2 Self‐Assessment of Insomnia and Subjective Adverse Events (Tables 2 and 3)

**TABLE 2:** Association between self‐assessment of insomnia and three drug classes.

Athens Insomnia Scale	Non‐BZDs (*n* = 88)	BZDs (*n* = 106)	ORAs (*n* = 117)	*p*
Total score ≧ 6	63 (71.6%)	55 (51.9%)	83 (70.9%)	0.0033**
< 6	25	51	34	
Sleep induction				
Score 2/3	21 (23.9%)	23 (21.7%)	32 (27.4%)	0.63
Score 0/1	67	83	85	
Awakenings during the night				
Score 2/3	15 (17.0%)	16 (15.1%)	25 (21.4%)	0.49
Score 0/1	73	90	92	
Final awakening earlier than desired				
Score 2/3	26 (29.5%)	25 (23.6%)	35 (29.9%)	0.52
Score 0/1	62	81	82	
Total sleep duration				
Score 2/3	12 (13.6%)	13 (12.3%)	20 (17.1%)	0.59
Score 0/1	76	93	97	
Overall sleep quality				
Score 2/3	13 (14.8%)	15 (14.2%)	28 (23.9%)	0.12
Score 0/1	75	91	89	
Sense of well‐being during the day				
Score 2/3	21 (23.9%)	20 (18.9%)	24 (20.5%)	0.69
Score 0/1	67	86	93	
Functioning during the day				
Score 2/3	22 (25.0%)	18 (17.0%)	30 2 (5.6%)	0.24
Score 0/1	66	88	87	
Sleepiness during the day				
Score 2/3	18 (20.5%)	20 (18.9%)	26 (22.2%)	0.85
Score 0/1	70	86	91	

*Note:* Fisher's exact test.

Abbreviations: BZDs, benzodiazepines; non‐BZDs, non‐benzodiazepines; ORAs, orexin receptor antagonists.

** *p* < 0.01.

**TABLE 3:** Association between subjective adverse events and three drug classes.

Adverse events	Total	Non‐BZDs (*n* = 88)	BZDs (*n* = 106)	ORAs (*n* = 117)	*p*
Total					
Yes (present)	97 (31.2%)	24 (27.3%)	39 (36.8%)	34 (29.1%)	0.30
No (absent)	214	64	67	83	
Lightheadedness					
Always/often	14 (4.5%)	6 (6.8%)	5 (4.7%)	3 (2.6%)	0.31
Occasional/none	297	82	101	114	
Vertigo					
Always/often	12 (3.9%)	5 (5.7%)	5 (4.7%)	2 (1.7%)	0.26
Occasional/none	299	83	101	115	
Head heaviness/headache					
Always/often	24 (7.7%)	6 (6.8%)	10 (9.4%)	8 (6.8%)	0.77
Occasional/none	287	82	96	109	
Nausea					
Always/often	5 (1.6%)	2 (2.3%)	1 (0.9%)	2 (1.7%)	0.86
Occasional/none	306	86	105	115	
Constipation					
Always/often	28 (8.9%)	7 (8.0%)	13 (12.3%)	8 (6.8%)	0.34
Occasional/none	283	81	93	109	
Abdominal pain					
Always/often	11 (3.5%)	3 (3.4%)	7 (6.6%)	1 (0.9%)	0.06
Occasional/none	300	85	99	116	
Dysgeusia					
Always/often	5 (1.6%)	1 (1.1%)	2 (1.9%)	2 (1.7%)	1.0
Occasional/none	306	87	104	115	
Thirst					
Always/often	32 (10.3%)	9 (10.2%)	10 (9.4%)	13 (11.1%)	0.97
Occasional/none	279	79	96	104	
Nightmares					
Always/often	32 (10.3%)	8 (9.1%)	13 (12.3%)	11 (9.4%)	0.77
Occasional/none	279	80	93	106	
Tremor					
Always/often	11 (3.5%)	2 (2.3%)	7 (6.6%)	2 (1.7%)	0.14
Occasional/none	300	86	99	115	
Abnormal behavior during sleep					
Always/often	18 (5.8%)	4 (4.5%)	5 (4.7%)	9 (7.7%)	0.59
Occasional/none	293	84	101	108	

*Note:* Fisher's exact test.

Abbreviations: BZDs, benzodiazepines; Non‐BZDs, non‐benzodiazepines; ORAs, orexin receptor antagonists.

The proportion of patients with an Athens Insomnia Scale score ≥ 6 was 71.6% for non‐BZDs, 51.9% for BZDs, and 70.9% for DORAs, with BZDs showing a significantly lower frequency (*p* = 0.0033). There were no significant differences among the three drug classes in the severity of any of the eight AIS items.

For the 11 self‐reported symptoms, two items (thirst and nightmares) were reported as “always or often” by more than 10% of patients; three items (constipation, head heaviness/headache, and abnormal behavior during sleep) by 5%–10%; and four items (lightheadedness, vertigo, abdominal pain, and tremor) by 3%–5%. No significant differences in the frequency of any adverse event were observed among the three drug classes.

Overall, the frequency of subjective adverse events was higher than the rates reported in package inserts.

### 3.3 Association Between Subjective Adverse Events and Concomitant Medications (Table 4)

**TABLE 4:** Association between subjective adverse events and concomitant medications.

Adverse events	ALs	ADs	APs	MSs	No concomitant medications	*p*
	(*n* = 130)	(*n* = 195)	(*n* = 47)	(*n* = 26)	(*n* = 58)	
Total						
Yes (present)	43 (33.1%)	72 (36.9%)	16 (34.0%)	13 (50.0%)	11 (19.0%)	0.042*
No (absent)	87	123	31	13	47	
Lightheadedness						
Always/often	9 (6.9%)	12 (6.2%)	1 (2.1%)	1 (3.8%)	1 (1.7%)	0.56
Occasional/none	121	183	46	25	57	
Vertigo						
Always/often	7 (5.4%)	8 (4.1%)	2 (4.3%)	2 (7.7%)	2 (3.4%)	0.86
Occasional/none	123	187	45	24	56	
Head heaviness/headache						
Always/often	12 (9.2%)	20 (10.3%)	6 (12.8%)	6 (23.1%)	0 (0%)	0.0053**
Occasional/none	118	175	41	20	58	
Nausea						
Always/often	2 (1.5%)	3 (1.5%)	0 (0%)	1 (3.8%)	0 (0%)	0.61
Occasional/none	128	192	47	25	58	
Constipation						
Always/often	8 (6.2%)	20 (10.3%)	4 (8.5%)	3 (11.5%)	4 (6.9%)	0.68
Occasional/none	122	175	43	23	54	
Abdominal pain						
Always/often	4 (3.1%)	6 (3.1%)	2 (4.3%)	3 (11.5%)	2 (3.4%)	0.30
Occasional/none	126	189	45	23	52	
Dysgeusia						
Always/often	2 (1.5%)	5 (2.6%)	0 (0%)	0 (0%)	0 (0%)	0.79
Occasional/none	128	190	47	26	58	
Thirst						
Always/often	17 (13.1%)	27 (13.8%)	5 (10.6%)	3 (11.5%)	2 (3.4%)	0.24
Occasional/none	113	168	42	23	56	
Nightmares						
Always/often	14 (10.8%)	26 (13.3%)	5 (10.6%)	4 (15.4%)	1 (1.7%)	0.080
Occasional/none	116	169	42	22	57	
Tremor						
Always/often	6 (4.6%)	5 (2.6%)	2 (4.3%)	2 (7.7%)	1 (1.7%)	0.45
Occasional/none	124	190	45	24	57	
Abnormal behavior during sleep						
Always/often	13 (10.0%)	15 (7.7%)	4 (8.5%)	3 (11.5%)	1 (1.7%)	0.27
Occasional/none	117	180	43	23	57	

*Note:* Fisher's exact test.

Abbreviations: ADs, antidepressants; ALs, anxiolytics; APs, antipsychotics; MSs, mood stabilizers.

* *p* < 0.05.

** *p* < 0.01.

Of the 341 patients, 283 were taking at least one concomitant psychotropic medication (antidepressants, anxiolytics, antipsychotics, or mood stabilizers), whereas 58 were not. Among the 11 adverse events, head heaviness/headache was significantly less frequent in patients not taking concomitant psychotropic medications.

### 3.4 Relationships Between Adverse Events and Multiple Independent Variables (Table 5)

**TABLE 5:** Multiple logistic regression analysis. Association between subjective adverse events and sex, age, hypnotic drug class, and DSM‐5‐TM classification.

	Odds ratio	95% CI		*p*
		Lower	Upper	
Sex (female/male)	1.96	1.11	3.45	0.020*
Age				
Under 40/over 59	2.29	1.10	4.78	0.027*
40 to 59/over 59	2.42	1.23	4.74	0.010*
Hypnotic drug classes				
BZDs/non‐BZDs	1.74	0.867	3.49	0.12
ORAs/non‐BZDs	1.22	0.610	2.42	0.58
DSM‐5‐TM classification				
NDDs/SWDs	2.12	0.538	8.36	0.28
SP/SWDs	6.14	1.51	24.9	0.011*
DDs/SWDs	3.21	1.03	10.0	0.045*
ADs/SWDs	5.29	1.57	17.8	0.0072**
PTSDs/SWDs	1.39	0.223	7.31	0.70

Abbreviations: ADs, anxiety disorders; BZDs, benzodiazepines; DDs, depressive disorders; NDDs, neurodevelopmental disorders; non‐BZDs, non‐benzodiazepines; ORAs, orexin receptor antagonists; PTSDs, trauma‐ and stress‐related disorders; SP, schizophrenia; SWDs, sleep–wake disorders.

* *p* < 0.05.

** *p* < 0.01.

The logistic regression model fit the data significantly better than the null model (chi‐square test, *p* = 0.0016), and all variance inflation factors were below 5. Female sex was significantly associated with higher odds of adverse events (*p* = 0.010). Patients aged ≥ 60 years had significantly lower odds of adverse events than those aged 40–59 years (*p* = 0.014) and those under 40 years (*p* = 0.026). Anxiety disorders (*p* = 0.0072), schizophrenia (*p* = 0.011), and depressive disorders (*p* = 0.045) were significantly associated with higher odds of adverse events. No associations were found between adverse events and hypnotic drug classes.

## 4 Discussion

A previous survey of US patients taking sleep medications revealed that the most important factors in drug selection were “efficacy” and “adverse events,” rather than cost [21]. A recent systematic review and meta‐analysis also demonstrated differences in efficacy and adverse events among hypnotics, even within the same drug class [22]. Discontinuation, switching, or addition of hypnotics shortly after initiation is often attributed to insufficient efficacy or intolerable adverse events. In a large Japanese cohort of approximately 240 000 patients, the 6‐month failure rate—defined as switching or adding another hypnotic—was 10.3%, with ramelteon showing the highest rate, followed by eszopiclone and suvorexant [23].

Long‐term use of hypnotics is common in clinical practice. In Italy, 73% of patients continued hypnotics for more than 1 year [24], and more than 50% of American cancer patients continued for over 5 years [25]. These findings suggest that once patients find a hypnotic regimen with acceptable efficacy and tolerability, they tend to continue it for extended periods. Therefore, understanding not only the efficacy but also the type and frequency of adverse events is essential for appropriate drug selection.

Although package inserts report low incidences of adverse events for hypnotics [11], real‐world clinical studies have consistently shown higher frequencies [12, 26, 27]. Package‐insert data are derived from controlled clinical trials with highly selected populations, whereas real‐world psychiatric outpatients often have multiple comorbidities, polypharmacy, and chronic hypnotic use. This discrepancy likely contributes to the higher frequency of subjective adverse events observed in routine practice.

In the present study, subjective symptoms such as thirst and nightmares were reported by more than 10% of patients, and heavy‐headedness/headache, constipation, and abnormal sleeping behavior by more than 5%, all of which exceeded the frequencies described in package inserts. Although vertigo is commonly associated with BZDs and non‐BZDs [8], its frequency did not differ among the three drug classes. Nightmares, frequently reported with DORAs [21, 26, 28, 29, 30], also showed no significant differences among drug classes in this study.

Importantly, the subjective adverse events identified in this study cannot be assumed to be directly caused by hypnotics. As psychiatric outpatients frequently have comorbid psychiatric and physical conditions and take multiple concomitant medications, patient‐reported symptoms may reflect these underlying factors rather than drug‐induced adverse reactions. Consistent with this, our multivariate analyses revealed that female sex, age < 60 years, and psychiatric diagnoses such as schizophrenia, depression, and anxiety disorders were associated with subjective adverse events, whereas hypnotic drug class was not. These findings suggest that patient‐related factors may play a greater role in the occurrence of subjective adverse events than the pharmacological class of hypnotics. This may also explain why real‐world adverse event frequencies are higher than those reported in clinical trials.

Recent evidence indicates that 66% of patients with subjective insomnia (Athens Insomnia Scale ≥ 6) do not have objective insomnia based on EEG data [31], highlighting the discrepancy between subjective perception and objective sleep parameters. Nevertheless, treatment decisions in clinical practice must rely on patients' subjective complaints and satisfaction. Therefore, routine assessment of subjective adverse events through interviews or questionnaires is essential for optimizing hypnotic therapy in real‐world settings.

## 5 Limitations

This study has several limitations. First, adverse events were assessed based on patients' subjective evaluations of their current hypnotic medications; adverse events associated with previously used hypnotics were not examined, introducing potential recall bias. Second, the study population consisted of heterogeneous psychiatric outpatients, many of whom had multiple psychiatric symptoms and were receiving combinations of antipsychotics, anxiolytics, antidepressants, and mood stabilizers. Because only a small number of patients were treated with hypnotics alone, concomitant medications and comorbid psychiatric conditions may have influenced the results. Third, although only patients taking a single hypnotic were included to minimize confounding by polypharmacy, the distribution of specific agents within each drug class—particularly the predominance of lemborexant among DORAs—may have affected class comparisons. Finally, although the study was conducted prospectively, the assessment itself was cross‐sectional, and therefore causal interpretations are limited.

## 6 Conclusions

In patients taking hypnotics, the frequency of subjective adverse events was higher than that stated in package inserts, underscoring a clear discrepancy between controlled trial data and real‐world clinical experience. These findings suggest that patient‐related factors such as psychiatric comorbidities, physical conditions, and concomitant medications may contribute more to the occurrence of adverse events than the hypnotic drug class itself. Therefore, routine assessment of subjective symptoms through interviews or questionnaires is essential for optimizing hypnotic therapy in everyday clinical practice.

## Author Contributions

Conception and design of study: Fumi Akasaka, Sotaro Sadahiro. Acquisition of data: Fumi Akasaka, Kuni Akasaka, Kazutake Okada. Analysis and interpretation of data: Yasuo Haruki, Naohito Yamaguchi. Writing – original drafting: Fumi Akasaka, Sotaro Sadahiro. Writing – review and editing: Yasuo Haruki, Sotaro Sadahiro.

## Funding

The authors have nothing to report.

## Ethics Statement

This study was conducted in accordance with the Ethical Guidelines for Medical Research Involving Human Subjects and was approved by the Ethics Committee of the Japanese Association of Neuropsychiatric Clinics (2023‐7).

## Consent

Informed consents were obtained from all participants before participating.

## Conflicts of Interest

The authors declare no conflicts of interest.

## Supporting information


**Data S1:** This file contains detailed item‐level frequencies and raw response distributions for all 11 subjective adverse events assessed in the study (lightheadedness, vertigo, head heaviness/headache, nausea, constipation, abdominal pain, dysgeusia, thirst, nightmares, tremors, and abnormal sleeping behavior). The dataset includes the full response matrix used for statistical analyses.

## Data Availability

All individual‐level data underlying the findings of this study are provided as a Supporting Information data file. No information that could identify individual participants is included.
